# Limited External Validation and Uncertain Clinical Utility of Prediction Models for Bacteriologically Negative Pulmonary Tuberculosis: A Systematic Review

**DOI:** 10.7759/cureus.116983

**Published:** 2026-09-26

**Authors:** Chuntao Zhai, Baosheng Zhao, Yu Yu, Yue Zhao, Xiaohua Yang, Yuzhen Liu, Ernest Mangantig

**Affiliations:** 1 Department of Community Health, Pusat Kanser Tun Abdullah Ahmad Badawi, Universiti Sains Malaysia, Penang, MYS; 2 Department of Thoracic Surgery, The First Affiliated Hospital of Henan Medical University, Xinxiang, CHN; 3 Department of Pediatrics, The First Affiliated Hospital of Henan Medical University, Xinxiang, CHN; 4 Life Science Research Center, The First Affiliated Hospital of Henan Medical University, Xinxiang, CHN; 5 Department of Respiratory and Critical Care Medicine, The First Affiliated Hospital of Henan Medical University, Xinxiang, CHN

**Keywords:** bacteriologically negative pulmonary tuberculosis, clinical utility, external validation, prediction model, systematic review

## Abstract

Prediction models for bacteriologically negative pulmonary tuberculosis (BN-PTB) may support diagnostic triage, but their external validity and clinical utility remain uncertain. We searched PubMed, Embase, Web of Science, and Scopus from database inception through December 31, 2025. The review was reported in accordance with the Preferred Reporting Items for Systematic Reviews and Meta-Analyses (PRISMA) 2020; risk of bias and applicability were assessed using Prediction model Risk Of Bias ASsessment Tool (PROBAST), and certainty of evidence was evaluated using an adaptation of Grading of Recommendations Assessment, Development and Evaluation (GRADE) for diagnostic test accuracy. Of 163 full-text reports, 160 were excluded because of population mismatch (n=63), target or reference-standard mismatch (n=52), methodological or design ineligibility (n=20), or model-use or reporting ineligibility (n=25). Three reports met the completed review eligibility criteria and contributed six distinct externally validated prediction models. Five models provided external-validation data sufficient for the extraction or reconstruction of model-specific 2×2 tables. The four Li QH et al. models had study-reported thresholds applied in the validation sample; for the Meng et al. model, the reported cutoff was 0.657, but its explicit linkage to the external-validation estimates was unclear. Consequently, the reconstructed Meng estimates were considered approximate. Model-specific sensitivities ranged from 0.877 to 0.982 and specificities from 0.864 to 0.986. No cross-model pooling was performed because four models shared one validation cohort and the models differed in predictor sets, threshold information, comparator spectra, and clinical settings. The Xu et al. nomogram reported an external C-index of 0.77 (95% confidence interval 0.68-0.86) but no fixed threshold or threshold-specific 2×2 data. Although several models showed favorable performance in their respective validation samples, the evidence was limited by shared validation data, disparate comparator spectra, incomplete threshold and calibration reporting, and the absence of repeated independent validation or prospective clinical-impact evaluation. The currently eligible evidence does not establish sufficient transportability or clinical utility for routine implementation.

## Introduction and background

Bacteriologically negative pulmonary tuberculosis (BN-PTB) remains a major diagnostic challenge within the global tuberculosis burden. In this review, BN-PTB was operationally defined as clinically suspected or clinically diagnosed pulmonary tuberculosis without a positive bacteriological result at the relevant diagnostic decision point. Bacteriological confirmation included smear microscopy, culture, or molecular testing when these tests were performed. Active tuberculosis could instead be established using a study-specific composite reference standard. Components could include compatible clinical and radiological findings, immunological evidence, exclusion of alternative diagnoses, treatment response, or a clinician-assigned final diagnosis. This was a review-specific eligibility definition, not a universal World Health Organization or national case definition. We therefore extracted each report's diagnostic criteria and treated cohorts with mixed or incompletely characterized bacteriological status as indirect evidence in the applicability assessment. BN-PTB may reflect paucibacillary disease, inadequate sputum specimens, or limited access to molecular assays such as Xpert *Mycobacterium tuberculosis*/rifampicin (Xpert MTB/RIF) assay [1-4]. An estimated 10.7 million people developed tuberculosis globally in 2024 [5]. Lack of bacteriological confirmation may delay treatment and infection-control measures in patients with tuberculosis [6,7]. Conversely, empirical treatment of patients without tuberculosis may cause drug toxicity and delay recognition of alternative pulmonary diseases.

Investigators have proposed multivariable diagnostic prediction models and machine-learning-assisted algorithms to support BN-PTB assessment. These tools may serve different clinical roles, including screening, triage, diagnostic support, isolation decisions, and treatment-initiation decisions. These roles are not interchangeable. Model interpretation therefore requires consideration of the intended clinical setting, target population, comparator spectrum, predictor measurement time point, prediction threshold, and reference standard. At a defined threshold, 2x2 data are required to estimate model-specific sensitivity, specificity, positive and negative likelihood ratios, and their uncertainty. Discrimination measures such as the area under the curve or C-index alone cannot provide these threshold-specific quantities. Existing approaches range from conventional logistic-regression scores [8,9] to least absolute shrinkage and selection operator models and deep-learning or computer-aided detection systems [10-18]. However, model development has outpaced independent external validation. Common limitations include spectrum bias, incomplete reference standards, internal validation only, unclear clinical-use pathways, and incomplete threshold-specific reporting [19,20].

It remains uncertain whether externally validated BN-PTB prediction models contain overlapping and clinically plausible predictors across populations or primarily reflect local case mix and measurement practices. We therefore assessed predictor overlap descriptively and harmonized predictors only when they represented the same clinical construct. This comparison was not considered a statistical assessment of predictor stability or generalizability. Clinical utility was defined as improvement in clinical decision-making, patient management, or patient outcomes within a specified care pathway. Discrimination, calibration, threshold-specific accuracy, likelihood ratios, and decision-curve analysis may describe model performance or potential decision relevance, but they do not independently demonstrate clinical utility. Because this evidence concerns diagnostic prediction rather than causal inference, methodological appraisal should be aligned with prediction-model guidance, including Transparent Reporting of a multivariable prediction model for Individual Prognosis or Diagnosis (TRIPOD) [19] and Prediction model Risk Of Bias ASsessment Tool (PROBAST) [20].

We conducted a systematic review in accordance with the Preferred Reporting Items for Systematic Reviews and Meta-Analyses (PRISMA) 2020 [21] to identify reports containing external-validation evaluations of multivariable prediction models for adults evaluated for BN-PTB. The objectives were to characterize each model's intended clinical role, target population, comparator spectrum, reference standard, predictors, threshold, and development-validation relationship; summarize model-specific external-validation performance when supported by the reported data; describe predictor overlap; appraise risk of bias and certainty of evidence; and determine whether the available evidence supports clinical implementation or indicates a need for further validation. Models were not assumed to be clinically interchangeable, and quantitative synthesis was considered only when clinically coherent and independent evaluations were available.

## Review

Material and methods

Study Design and Reporting Standards

This systematic review was reported in accordance with the PRISMA 2020 statement [21]. The reporting and interpretation of prediction-model studies were informed by the Transparent Reporting of a multivariable prediction model for Individual Prognosis or Diagnosis (TRIPOD) statement [19] and the PROBAST [20]. Where applicable, artificial intelligence and machine-learning studies were considered in relation to the TRIPOD+AI extension [22], and diagnostic accuracy reporting was considered in relation to Standards for Reporting Diagnostic Accuracy Studies (STARD) 2015 [23].

The review was registered with PROSPERO (CRD420251168396) [24]. During peer-review revision, the synthesis plan was amended by replacing exploratory cross-model pooling with model-specific reporting. This change was made because the included reports evaluated distinct prediction models with different predictor sets, thresholds, comparator spectra, and clinical settings. In addition, four models from one report were evaluated in the same validation cohort and therefore did not provide independent estimates. This post-registration analytical change is reported transparently.

Search Strategy

We systematically searched PubMed, Embase, Web of Science, and Scopus on March 7, 2026, covering records from database inception through December 31, 2025. The search combined controlled vocabulary and free-text terms related to *Mycobacterium tuberculosis*, bacteriologically negative pulmonary tuberculosis, smear-negative pulmonary tuberculosis, diagnostic prediction models, machine learning, artificial intelligence, and diagnostic accuracy. Database-specific search strings were adapted to each platform. The searched databases, execution date, and coverage are summarized in Table 3 in the Appendices; the complete database-specific search strings are presented immediately below the table.

No study registers were searched. PROSPERO was used solely to register this systematic review and was not used as an information source for identifying primary studies. All retrieved records were imported into Mendeley Reference Manager version 2.139.0 (Elsevier Ltd, Kidlington, United Kingdom), and records identified as duplicates using its duplicate-management function were removed before screening.

No language, publication-type, or publication-status filters were encoded in the retained database search strategies. The documented information sources comprised the four bibliographic databases listed above; no separate supplementary-search record was available for inclusion in this revision. No additional manual duplicate-review rule was claimed because the retained Mendeley documentation records use of the duplicate-management function but does not preserve a separate manual matching rule.

Study Selection and Eligibility Criteria

During revision, Chuntao Zhai and Yu Yu independently rechecked the title-and-abstract and full-text screening decisions. Disagreements were resolved through discussion, and unresolved eligibility decisions were adjudicated by Xiaohua Yang. The 405 records identified during pre-screening as potentially ineligible were manually reviewed against the eligibility criteria before exclusion; no record was excluded solely by an automation tool. Because this verification occurred before formal title-and-abstract screening, these records are reported as removed before screening in Figure 1. The retained screening records did not preserve the original pre-screening flagging rule or software source.

Reports were eligible if they evaluated adults aged 18 years or older in populations relevant to suspected or diagnosed BN-PTB and contained an external-validation evaluation of a multivariable diagnostic prediction model. Eligible models included conventional regression-based scores, penalized regression models, nomograms, and machine-learning-assisted algorithms, provided that they generated an individual-level diagnostic prediction or risk estimate relevant to BN-PTB. External validation was required because the review question concerned model performance beyond the data used for development and potential transportability to new participants; however, nominal external validation alone was not assumed to establish generalizability.

Reports were retained in the systematic review if they provided extractable external-validation performance information, including sensitivity, specificity, threshold-specific 2x2 data, area under the curve (AUC), or C-index. Availability of threshold-specific 2x2 data was not required for inclusion in the systematic review. Such data were required for calculating model-specific sensitivity, specificity, and likelihood ratios and for inclusion in the corresponding forest plots.

All distinct eligible models reported within an included report were retained as separate model-level units. We did not select only the best-performing model from each report. For models with a fixed threshold explicitly linked to the external-validation analysis, the reported threshold was used for model-specific calculations. The four Li QH et al. models met this condition. Meng et al. reported a cutoff of 0.657, but the report did not explicitly link that cutoff to the external-validation estimates; its model-specific 2x2 table was therefore reconstructed from the reported validation totals and performance measures rather than attributed unequivocally to that cutoff. No threshold was selected post hoc on the basis of the highest sensitivity, specificity, AUC, or C-index. Models reporting only an AUC or C-index without a fixed threshold or threshold-specific 2x2 data were retained for narrative and tabular synthesis, but no 2x2 table was reconstructed from discrimination statistics.

At the full-text stage, each excluded report was assigned one primary reason using a mutually exclusive hierarchical framework applied during review implementation. The first applicable reason was recorded in the following order: (1) population mismatch; (2) target or reference-standard mismatch; (3) methodological or design ineligibility; and (4) model-use or reporting ineligibility. This operational hierarchy was not specified in the PROSPERO record and is therefore reported as a post-registration implementation decision rather than as a prospectively prespecified criterion.

Population mismatch included reports involving participants younger than 18 years or populations outside the intended adult BN-PTB diagnostic context, including HIV-dominant, pregnancy-specific, or other condition-restricted cohorts that did not represent the target clinical pathway. Target or reference-standard mismatch included reports that predicted microbiological test positivity, such as smear, culture, polymerase chain reaction (PCR), or Xpert positivity, rather than the differential diagnosis of BN-PTB supported by an eligible clinical or composite reference standard.

Methodological or design ineligibility included reports that did not evaluate an eligible multivariable prediction model, used development-only or internal random-split assessment without an eligible external validation, reported duplicate or overlapping evidence without a distinct eligible validation, or otherwise failed the review's design requirements. PROBAST risk-of-bias judgments were not used as an exclusion criterion.

Model-use or reporting ineligibility included models developed solely for airborne-isolation decisions, population-level active case finding, or standalone artificial-intelligence or computer-aided-detection threshold testing outside the intended symptomatic diagnostic-support pathway, as well as reports without extractable eligible external-validation performance information.

Data Extraction and Quality Assessment

During revision, Yue Zhao extracted the report-level and model-level data, and Xiaohua Yang independently checked all extracted fields against the source articles. Discrepancies were resolved through discussion, with Chuntao Zhai making the final determination when required. This was an extraction-plus-independent-verification process rather than duplicate independent extraction.

Extracted information included report characteristics, participant characteristics, target population, comparator spectrum, reference standard, model name, intended clinical role, predictor variables, modeling approach, validation setting, reported classification threshold, 2x2 data, sensitivity, specificity, likelihood ratios, and discrimination measures. Report-level and model-level information was distinguished throughout data extraction and synthesis.

When a report contained multiple distinct models, each model was extracted and presented separately. Source-reported target orientation was preserved in the extraction table. For the review-standardized model-specific calculations, BN-PTB was treated as the positive target condition. Any reorientation of source-reported contingency data was documented. Missing threshold-specific information was recorded as not reported and was not inferred from an AUC or C-index.

Risk of bias and applicability were assessed using PROBAST [20] at the level of each distinct prediction model. When a report included both model development and external validation, these evaluations were considered separately. The applicable signaling questions were rated as yes, probably yes, probably no, no, or no information; development-only questions were marked not applicable for validation evaluations. Domain-level risk of bias was judged for participants, predictors, outcome, and analysis, and applicability was judged separately for participants, predictors, and outcome. Applicability was evaluated for diagnostic support in adults with suspected BN-PTB at the initial diagnostic assessment, before a definitive treatment decision and before treatment-response information was available. Source-supported rationales and complete signaling-question assessments are presented in Tables 4 and 5 in the Appendices, respectively. Chuntao Zhai completed the initial assessment, and Xiaohua Yang independently reassessed all judgments during revision. Disagreements were resolved through discussion; Yu Yu participated in consensus adjudication when required.

Certainty of evidence was evaluated using the Grading of Recommendations Assessment, Development and Evaluation (GRADE) framework adapted for diagnostic test accuracy (GRADE-DTA) [25]. GRADE-DTA assessments were applied to each relevant model-outcome evidence body rather than used as definitive ratings of individual reports. The evidence profile considered risk of bias, inconsistency, indirectness, imprecision, and publication bias. Because each model-outcome evidence body contained only one external-validation evaluation, inconsistency and publication bias across studies were classified as not assessable. Chuntao Zhai completed the initial assessment, and Xiaohua Yang independently reassessed the evidence profile during revision. Final judgments were established through consensus, with Yu Yu participating in adjudication when required. The model- and outcome-specific GRADE-DTA evidence profile is presented in Table 6 in the Appendices.

Data Synthesis and Statistical Analysis

The primary synthesis was conducted at the model level. Three reports contributed six distinct externally validated prediction models. Five models provided external-validation data sufficient for extraction or reconstruction of model-specific 2x2 tables. The four Li QH et al. [26] models had study-reported thresholds applied in the validation sample. For the Meng et al. [10] model, the reported cutoff was 0.657, but its explicit linkage to the external-validation estimates was unclear. The sixth model reported an external C-index but did not provide a fixed classification threshold or threshold-specific 2x2 data.

For the five models with data sufficient for extraction or reconstruction of 2x2 tables, sensitivity and specificity were calculated separately for each model and presented with exact binomial 95% confidence intervals. The four Li QH et al. tables were extracted from the detailed validation results. The Meng et al. table was reconstructed from the reported validation totals and performance measures. Accordingly, the reconstructed 2×2 table and derived likelihood ratios were treated as approximate and were not attributed unequivocally to the reported cutoff of 0.657. Model-specific positive and negative likelihood ratios were derived from these 2x2 tables. Their 95% confidence intervals were calculated on the logarithmic scale using standard errors derived from the cell counts. None of the five 2x2 tables contained a zero cell, so no continuity correction was required. Likelihood ratios were reported separately for each model and were not pooled.

Model-specific sensitivity and specificity estimates were displayed in forest plots without pooled summary estimates or summary diamonds. Four models reported by Li QH et al. [26] were evaluated in the same validation cohort and were therefore statistically dependent. The model reported by Meng et al. [10] was evaluated in a separate cohort with a different comparator spectrum. The model reported by Xu et al. [18] was retained in the systematic review but was not included in the sensitivity and specificity forest plots because only an external C-index was reported.

No cross-model meta-analysis was performed because the available estimates represented different prediction models, thresholds, predictor sets, comparator populations, and clinical settings. The four models evaluated in the shared Li QH et al. cohort could not be treated as independent studies. Consequently, no pooled sensitivity, pooled specificity, hierarchical summary receiver operating characteristic model, I-squared statistic, Cochran's Q test, pooled likelihood ratio, or pooled Fagan analysis was calculated. Meta-regression and formal publication-bias analyses were also not performed because only three reports were included and no statistically coherent pooled analysis was undertaken.

All calculations were implemented using the reproducible base-R script and model-level dataset prepared for this revision. Analyses were performed using R version 4.5.3 (R Foundation for Statistical Computing, Vienna, Austria) within RStudio version 2026.01.1 Build 403 (Posit Software, PBC, Boston, MA, USA). Data and code availability: The source-aligned model-level dataset and reproducible base-R script used for the model-specific calculations are available from the corresponding author upon reasonable request.

Results

Study Selection

Following deduplication and screening, 163 reports underwent full-text eligibility assessment (Figure 1). Each excluded report was assigned the first applicable primary reason in the implementation-stage hierarchy described in the Methods. Of the 163 full-text reports, 160 were excluded: 63 for population mismatch, 52 for target or reference-standard mismatch, 20 for methodological or design ineligibility, and 25 for model-use or reporting ineligibility.

**Figure 1:**
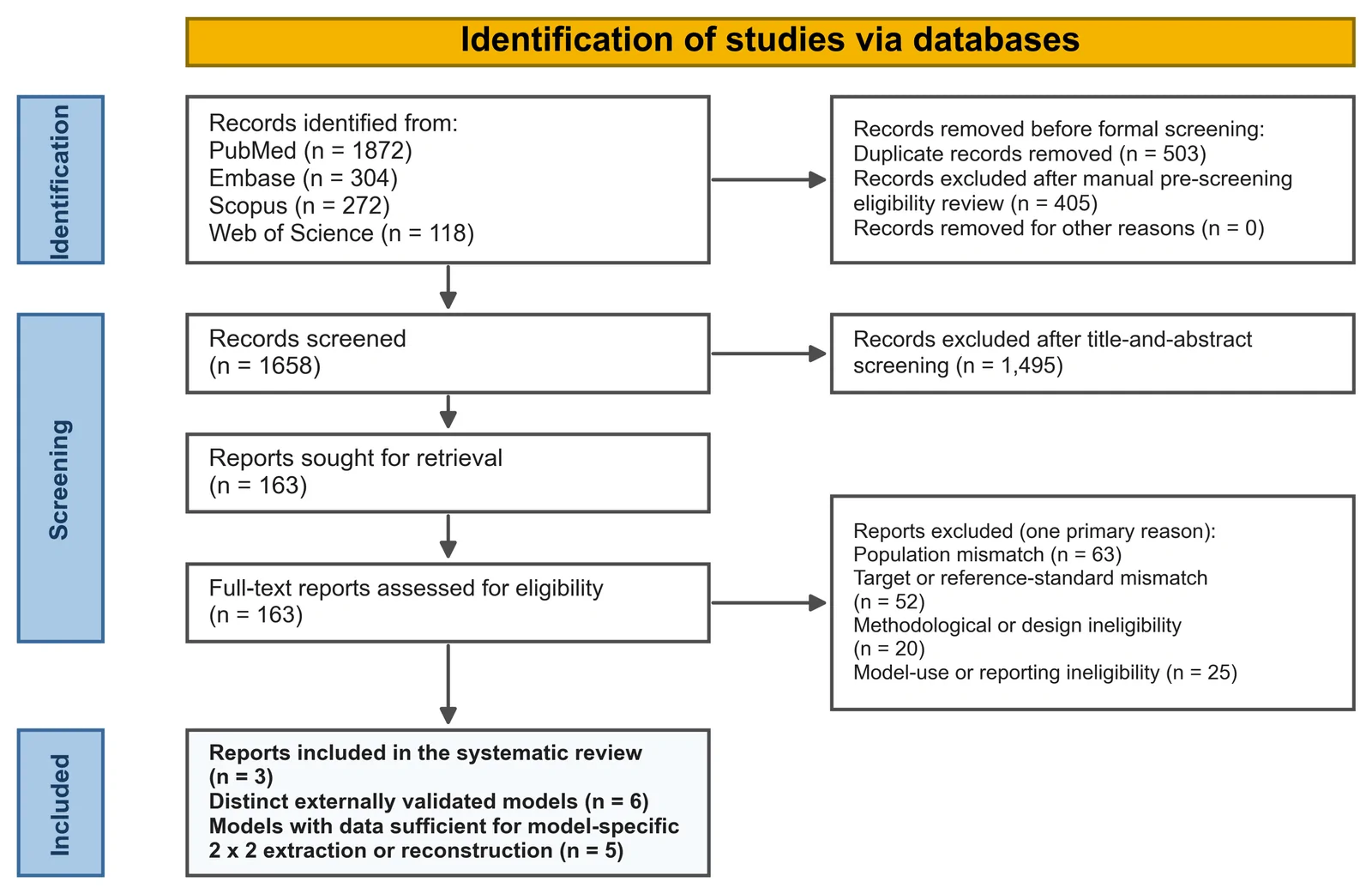
PRISMA 2020 flow diagram of study identification and selection The database searches identified 2,566 records. Before formal screening, 503 duplicate records and 405 records found to be ineligible during manual pre-screening review were removed. Of the 1,658 records screened, 1,495 were excluded after title-and-abstract screening, including 18 records previously classified as not retrieved that were subsequently confirmed to be ineligible at this stage. All 163 reports sought for retrieval were assessed for full-text eligibility. After 160 full-text exclusions, three reports evaluating six distinct externally validated models were included; five models provided data sufficient for extraction or reconstruction of model-specific 2×2 tables. PRISMA: Preferred Reporting Items for Systematic Reviews and Meta-Analyses.

Three reports met the completed review eligibility criteria: Li QH et al., 2012 [26]; Meng et al., 2021 [10]; and Xu et al., 2023 [18]. These reports contributed six distinct externally validated prediction models. Li QH et al. [26] contributed four models: clinical-radiographic (CR), clinical-radiographic-radionuclide (CRE), clinical-radiographic-pathological (CRP), and clinical-radiographic-radionuclide-pathological (CREP). Meng et al. [10] contributed one least absolute shrinkage and selection operator (LASSO)-logistic model, and Xu et al. [18] contributed one five-variable nomogram.

Five of the six models, comprising the four Li QH et al. models and the Meng et al. model, provided external-validation data sufficient for extraction or reconstruction of model-specific 2x2 tables. The four Li QH et al. models had study-reported thresholds applied in the validation sample. Because the report did not explicitly link the cutoff of 0.657 to the external-validation estimates, the reconstructed 2×2 table and derived likelihood ratios are approximate and cannot be attributed unequivocally to that cutoff. Their model-specific sensitivity and specificity estimates are presented in Figure 2 without pooled summary estimates. Xu et al. [18] reported an external C-index but did not provide a fixed threshold or threshold-specific 2x2 data. This model was retained in the systematic review and summarized narratively and in Table 1, but it was not included in the sensitivity and specificity forest plots. The characteristics, clinical roles, comparator spectra, reference standards, thresholds, model-specific performance measures, and external-validation settings of all six models are summarized in Table 1.

**Figure 2:**
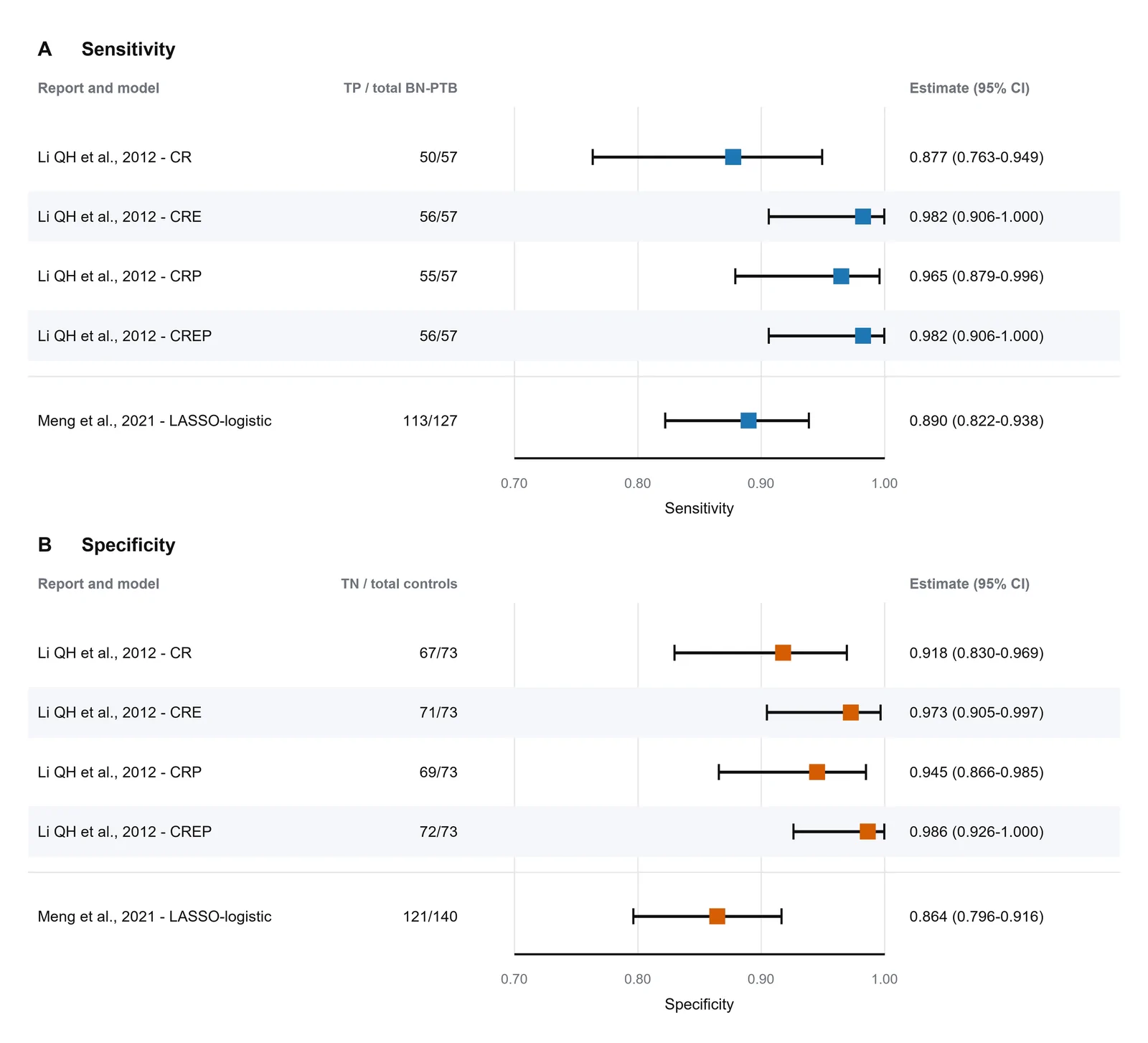
Model-specific external-validation sensitivity and specificity of BN-PTB prediction models Forest plots show model-specific sensitivity (Panel A) and specificity (Panel B) in external validation for five models with extractable or reconstructable 2×2 data. Squares indicate point estimates, and horizontal lines indicate exact binomial 95% confidence intervals. Panel A reports true positives among patients with BN-PTB; Panel B reports true negatives among controls. The CR, CRE, CRP, and CREP models reported by Li QH et al. [26] used study-reported thresholds in the same validation cohort; their estimates are statistically dependent. For the Meng et al. LASSO-logistic model [10], the reported cutoff was 0.657 but was not explicitly linked to the external-validation estimates. Its displayed 2×2 table was reconstructed from reported validation totals and performance measures; therefore, the table and derived likelihood ratios are approximate and cannot be attributed unequivocally to that cutoff. No pooled estimate, summary diamond, heterogeneity statistic, or HSROC model was calculated. The Xu et al. nomogram [18] is summarized in Table 1 but omitted because only an external C-index was reported, without threshold-specific 2×2 data. BN-PTB, bacteriologically negative pulmonary tuberculosis; CI, confidence interval; CR, clinical-radiographic score; CRE, clinical-radiographic-radionuclide score; CRP, clinical-radiographic-pathological score; CREP, clinical-radiographic-radionuclide-pathological score; HSROC, hierarchical summary receiver operating characteristic; LASSO, least absolute shrinkage and selection operator.

**Table 1:** Characteristics and model-specific external-validation performance of six prediction models from three reports All six eligible models are presented separately; no post hoc best-model selection or cross-model pooling was performed. The four models reported by Li QH et al. [26] share the same external-validation cohort, so their estimates are statistically dependent. For Li QH et al. [26], data were extracted from the detailed results because the surrounding narrative contained internally inconsistent numerators; source-reported sarcoidosis-positive results were reoriented as tuberculosis-positive for this review. For Meng et al. [10], the 2×2 table was reconstructed from the reported validation totals and sensitivity and specificity and was cross-checked against reported predictive values after rounding. Because the report did not explicitly link the cutoff of 0.657 to the external-validation estimates, the reconstructed 2×2 table and derived likelihood ratios are approximate and cannot be attributed unequivocally to that cutoff. Xu et al. [18] did not report a fixed threshold or threshold-specific 2×2 data. AFB, acid-fast bacilli; AUC, area under the curve; BN-PTB, bacteriologically negative pulmonary tuberculosis; CI, confidence interval; CR, clinical-radiographic score; CRE, clinical-radiographic-radionuclide score; CRP, clinical-radiographic-pathological score; CREP, clinical-radiographic-radionuclide-pathological score; IGRA, interferon-gamma release assay; NLR, negative likelihood ratio; NR, not reported; PLR, positive likelihood ratio; PTB, pulmonary tuberculosis; TN, true negative; TP, true positive.

Report/model	Validation cohort, target/comparator, and reference standard	Intended clinical role	Threshold and data use	TP/FN/TN/FP	Sensitivity (95% CI)	Specificity (95% CI)	PLR (95% CI)	NLR (95% CI)
Li QH et al. [26] / CR	n=130: 73 sarcoidosis and 57 sputum-negative TB; comparator: Sarcoidosis; reference standard: Study-specific TB and sarcoidosis criteria incorporating negative sputum bacteriology, biopsy findings, treatment response, and exclusion of granulomatous infection for sarcoidosis	Differential diagnosis of sarcoidosis versus sputum-negative TB	9; Selected during development and applied in the new validation sample	50/7/67/6	0.877 (95% CI 0.763-0.949)	0.918 (95% CI 0.830-0.969)	10.67 (95% CI 4.93-23.11)	0.134 (95% CI 0.067-0.269)
Li QH et al. [26] / CRE	n=130: 73 sarcoidosis and 57 sputum-negative TB; comparator: Sarcoidosis; reference standard: Study-specific TB and sarcoidosis criteria incorporating negative sputum bacteriology, biopsy findings, treatment response, and exclusion of granulomatous infection for sarcoidosis	Differential diagnosis of sarcoidosis versus sputum-negative TB	17; Selected during development and applied in the new validation sample	56/1/71/2	0.982 (95% CI 0.906-1.000)	0.973 (95% CI 0.905-0.997)	35.86 (95% CI 9.14-140.73)	0.018 (95% CI 0.003-0.126)
Li QH et al. [26] / CRP	n=130: 73 sarcoidosis and 57 sputum-negative TB; comparator: Sarcoidosis; reference standard: Study-specific TB and sarcoidosis criteria incorporating negative sputum bacteriology, biopsy findings, treatment response, and exclusion of granulomatous infection for sarcoidosis	Differential diagnosis of sarcoidosis versus sputum-negative TB	18; Selected during development and applied in the new validation sample	55/2/69/4	0.965 (95% CI 0.879-0.996)	0.945 (95% CI 0.866-0.985)	17.61 (95% CI 6.78-45.72)	0.037 (95% CI 0.010-0.145)
Li QH et al. [26] / CREP	n=130: 73 sarcoidosis and 57 sputum-negative TB; comparator: Sarcoidosis; reference standard: Study-specific TB and sarcoidosis criteria incorporating negative sputum bacteriology, biopsy findings, treatment response, and exclusion of granulomatous infection for sarcoidosis	Differential diagnosis of sarcoidosis versus sputum-negative TB	22; Selected during development and applied in the new validation sample	56/1/72/1	0.982 (95% CI 0.906-1.000)	0.986 (95% CI 0.926-1.000)	71.72 (95% CI 10.24-502.48)	0.018 (95% CI 0.003-0.124)
Meng et al. [10] / LASSO-logistic model	Performance text: n=267, comprising 127 PTB and 140 non-TB controls; Table 1 separately labels an indicator cohort of n=217; comparator: Non-TB disease controls; reference standard: WS 288-2017 final diagnosis by two experienced pulmonologists independently; validation additionally included etiologically confirmed PTB	Diagnostic support for PTB among clinically suspected patients versus non-TB disease controls	0.657 reported; explicit linkage to the external-validation estimates was unclear	113/14/121/19	0.890 (95% CI 0.822-0.938)	0.864 (95% CI 0.796-0.916)	6.56 (95% CI 4.30-10.00)	0.128 (95% CI 0.077-0.210)
Xu et al. [18] / Five-variable nomogram	n=134 recruited; 15 excluded for missing variables; n=119 analyzed: 77 AFB-negative/IGRA-positive TB and 42 pneumonia; comparator: Pneumonia; reference standard: Study-specific AFB-negative TB criteria using negative bacteriology plus imaging/clinical response; pneumonia per WS 382-2012 with specified exclusions	Differentiation of AFB-negative/IGRA-positive TB from pneumonia	NR; No fixed threshold reported	NR	NR	NR	NR	NR

Methodological Quality and Certainty of Evidence

The three included reports contained six distinct prediction models and 12 PROBAST evaluation units when development and external validation were assessed separately. In the final consensus assessment, all 12 evaluations were judged at high overall risk of bias. The Li evaluations were limited by case-control sampling, lack of reported blinding, mixed reference-standard pathways, data-driven predictor and threshold selection, suboptimal missing-data methods, and incomplete assessment of calibration and optimism. The Meng development evaluation used a prospective consecutive cohort and prespecified admission-time predictors but remained at high overall risk because of limited effective sample size relative to candidate predictors and potential incorporation or review bias in the clinical reference diagnosis; its external validation also mixed clinically diagnosed and etiologically confirmed pulmonary tuberculosis. The Xu evaluations were limited by selective complete-case samples, univariable screening, small development and validation samples, incorporation of white blood cell count into both the predictor set and pneumonia definition, and differential outcome determination. Applicability concerns were high for all Li and Xu evaluations and for the Meng external validation. The associated model-specific external-validation evidence bodies were rated very low certainty in the final GRADE-DTA profile, and no direct evidence of patient-important clinical impact was identified.

**Table 2:** PROBAST risk-of-bias and applicability judgments for 12 model-evaluation units Development and external-validation evaluations were assessed separately for each distinct model. P, participants; Pr, predictors; O, outcome; A, analysis; app, concern regarding applicability; BN-PTB, bacteriologically negative pulmonary tuberculosis; PROBAST, Prediction model Risk Of Bias ASsessment Tool; ROB, risk of bias. Applicability was judged for diagnostic support in adults with suspected BN-PTB at the initial diagnostic assessment, before a definitive treatment decision and before treatment-response information was available. Source-supported rationales and complete signaling-question assessments are presented in Tables 4 and 5 in the Appendices, respectively.

Study	Model	Evaluation	P ROB	Pr ROB	O ROB	A ROB	Overall ROB	P app	Pr app	O app	Overall app
Li QH et al.	CR	Development	High	High	High	High	High	High	Low	High	High
Li QH et al.	CR	External validation	High	Unclear	High	High	High	High	Low	High	High
Li QH et al.	CRE	Development	High	High	High	High	High	High	High	High	High
Li QH et al.	CRE	External validation	High	Unclear	High	High	High	High	High	High	High
Li QH et al.	CRP	Development	High	High	High	High	High	High	High	High	High
Li QH et al.	CRP	External validation	High	Unclear	High	High	High	High	High	High	High
Li QH et al.	CREP	Development	High	High	High	High	High	High	High	High	High
Li QH et al.	CREP	External validation	High	Unclear	High	High	High	High	High	High	High
Meng et al.	LASSO-logistic	Development	Low	Low	High	High	High	Low	Low	Low	Low
Meng et al.	LASSO-logistic	External validation	Low	Low	High	Unclear	High	High	Low	High	High
Xu et al.	Nomogram	Development	High	Low	High	High	High	High	Low	High	High
Xu et al.	Nomogram	External validation	High	Low	High	High	High	High	Low	High	High

Model-Specific External-Validation Performance

Five of the six models provided external-validation data sufficient for extraction or reconstruction of model-specific 2x2 tables and were therefore included in the model-specific forest plots (Figure 2). The four Li QH et al. models had study-reported thresholds applied in the validation sample. For the Meng et al. model, the reported cutoff was 0.657, but it was not explicitly linked to the external-validation estimates; consequently, the reconstructed 2×2 results and derived likelihood ratios should be interpreted as approximate and cannot be attributed unequivocally to that cutoff. Across the five displayed models, sensitivity ranged from 0.877 to 0.982 and specificity ranged from 0.864 to 0.986. Figure 2 presents the sensitivity and specificity of each model with exact binomial 95% confidence intervals, without a pooled summary estimate or summary diamond.

The CR, CRE, CRP, and CREP models reported by Li QH et al. [26] were evaluated in the same external validation cohort and therefore provided statistically dependent estimates. The LASSO-logistic model reported by Meng et al. [10] was evaluated in a separate cohort with a different comparator spectrum. Because these were distinct models with different predictor sets, threshold information, comparator populations, and clinical settings, the estimates were not pooled and should not be interpreted as direct comparative rankings.

The five-variable nomogram reported by Xu et al. [18] was retained in the systematic review and is summarized in Table 1. It was not included in Figure 2 because the report provided an external C-index without a fixed classification threshold or threshold-specific 2x2 data. Model-specific positive and negative likelihood ratios for the five models with 2 x 2 data are also reported in Table 1.

Predictor Overlap and Model Specification Heterogeneity

Predictor overlap across the six models is shown in Figure 3. The four related models reported by Li QH et al. [26] had nested predictor sets: CR contained six predictors, CRE added one radionuclide-imaging predictor, CRP added two pathological predictors, and CREP contained all nine predictors. The Meng LASSO-logistic model contained seven predictors and the Xu nomogram contained five. Six predictors were shared across all four Li models, two additional pathological predictors were shared by CRP and CREP, and one additional radionuclide-imaging predictor was shared by CRE and CREP. Six predictors were unique to Meng et al. [10] and four were unique to Xu et al. [18]. Age was the only predictor shared across different reports, occurring in the Meng and Xu models; no predictor was present in all six models. Age is nonspecific, and its contribution may vary with case mix. These descriptive overlaps do not establish predictor stability or transportability.

**Figure 3:**
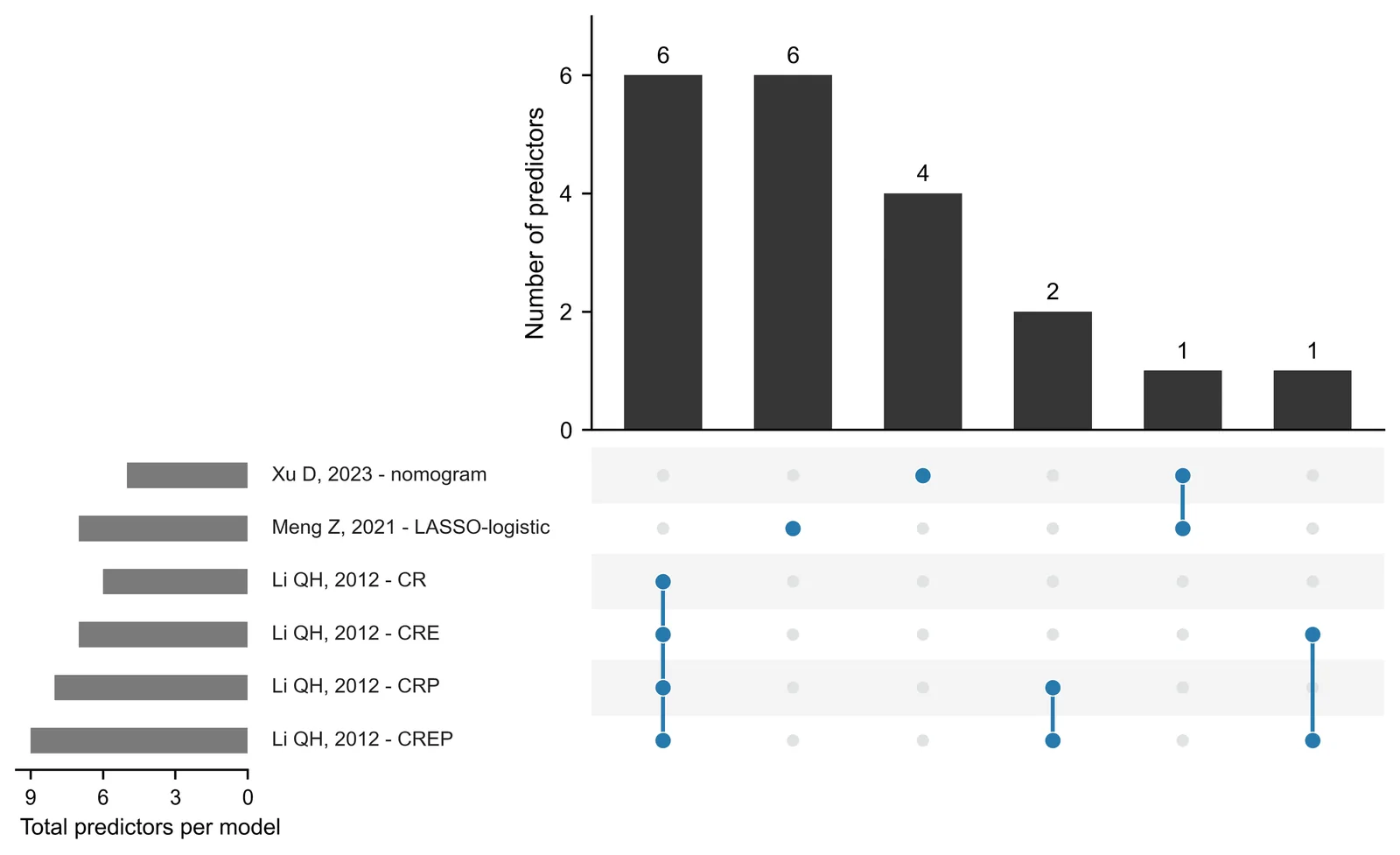
Final-predictor overlap across six externally validated BN-PTB prediction models UpSet plot showing the composition and exact overlap of final predictors across six externally validated BN-PTB prediction models reported in three reports. Horizontal bars show the total number of predictors in each model, while vertical bars show the number of predictors in each exact intersection. Connected blue dots identify the models contributing to each intersection. Six clinical-radiographic predictors were shared by the four models reported by Li QH et al. [26]. Six predictors were unique to the LASSO-logistic model reported by Meng et al. [10], and four were unique to the nomogram reported by Xu et al. [18]. Two pathological predictors were shared by CRP and CREP. Age was the only predictor shared across different reports, occurring in the Meng and Xu models. ECT manifestations were shared by CRE and CREP. No predictor was shared across all six models. The four Li models were evaluated in the same external validation cohort and therefore do not represent independent model evaluations. Predictor names were harmonized at the variable-concept level; categorical levels for lesion location and ECT manifestations were counted as single predictor variables. BN-PTB, bacteriologically negative pulmonary tuberculosis; CR, clinical-radiographic score; CRE, clinical-radiographic-radionuclide score; CRP, clinical-radiographic-pathological score; CREP, clinical-radiographic-radionuclide-pathological score; ECT, emission computed tomography; LASSO, least absolute shrinkage and selection operator.

Discussion

This systematic review identified three reports describing six distinct externally validated prediction models for BN-PTB. Five models provided external-validation data sufficient for extraction or reconstruction of model-specific 2x2 tables and calculation of model-specific sensitivity, specificity, positive likelihood ratios, and negative likelihood ratios. The four Li QH et al. models had study-reported thresholds applied in the validation sample. For the Meng et al. model, the reported cutoff was 0.657, but it was not explicitly linked to the external-validation estimates; consequently, the reconstructed 2×2 results and derived likelihood ratios should be interpreted as approximate and cannot be attributed unequivocally to that cutoff. The nomogram reported by Xu et al. [18] provided an external C-index but no fixed-threshold 2x2 data.

Several models showed favorable diagnostic performance in their respective validation samples. However, the models differed in predictor composition, classification thresholds, comparator populations, intended diagnostic roles, and clinical settings. Furthermore, the CR, CRE, CRP, and CREP models reported by Li QH et al. [26] were evaluated in the same external validation cohort and therefore did not represent independent validation samples. Cross-model pooling or pooling of models evaluated in the same cohort would consequently produce a summary estimate without a clearly defined clinical interpretation. We therefore retained all six eligible models and presented their validation performance at the model level without calculating pooled accuracy estimates.

Taken together, the currently eligible external-validation evidence suggests that some BN-PTB prediction models may have diagnostic potential within the specific populations in which they were evaluated. Nevertheless, the small number of independent reports, substantial differences in clinical settings and comparator spectra, and absence of repeated independent validation of the same model limit conclusions regarding transportability and routine clinical implementation. These conclusions apply to models meeting the completed review eligibility criteria and not to the totality of published BN-PTB prediction-model research.

Clinical and Methodological Heterogeneity

The heterogeneity among the included reports was clinical and methodological rather than a quantity summarized statistically. Li QH et al. [26] evaluated four related models for distinguishing sputum-negative pulmonary tuberculosis from sarcoidosis. Meng et al. [10] evaluated a LASSO-logistic model in a validation cohort containing pulmonary tuberculosis and non-tuberculosis disease controls. Xu et al. [18] evaluated a five-variable nomogram in patients with bacteriologically negative active pulmonary tuberculosis and pneumonia controls. These comparator populations represent different diagnostic problems and clinical spectra.

Sensitivity, specificity, and likelihood ratios are conditional on the population, reference standard, classification threshold, and distribution of alternative diagnoses. Differences in model-specific performance may therefore reflect differences in case mix and comparator spectrum rather than differences in the intrinsic quality of the models. The estimates displayed in Figure 2 should consequently be interpreted as descriptive validation results and should not be used to rank the models.

Dependence among model estimates was another important consideration. The four models reported by Li QH et al. [26] were assessed in the same validation cohort. Their estimates are correlated because they were derived from the same participants and outcomes. Treating them as independent studies would underestimate uncertainty and give disproportionate weight to a single report. A conventional meta-analysis was therefore not considered appropriate.

The models also differed substantially in predictor composition and modeling approach. This variation may reflect differences in intended clinical role, available measurements, comparator diseases, and local healthcare settings. The currently eligible evidence is insufficient to establish a reproducible predictor set that generalizes across populations. A stable predictor architecture would require head-to-head evaluation and repeated external validation of the same models in independent, clinically representative cohorts.

Methodological Quality and Reporting Completeness

The PROBAST and GRADE-DTA assessments were independently checked by a second assessor. These assessments considered participant selection, comparator spectrum, analytical methods, applicability to the intended clinical pathway, and completeness of performance reporting. The domain-level and overall judgments are summarized in Table 2. Source-supported PROBAST rationales, complete signaling-question assessments, and the model- and outcome-specific GRADE-DTA evidence profile are presented in Tables 4-6 in the Appendices.

Five models provided external-validation information sufficient for extraction or reconstruction of model-specific 2x2 tables. The four Li QH et al. models had study-reported thresholds applied in the validation sample. For the Meng et al. model, the 2×2 table was reconstructed from the reported validation totals and performance measures. Because the reported cutoff of 0.657 was not explicitly linked to those external-validation estimates, the reconstructed 2×2 results and derived likelihood ratios should be interpreted as approximate and cannot be attributed unequivocally to that cutoff. By contrast, the model reported by Xu et al. [18] provided an external C-index without a fixed classification threshold or threshold-specific contingency data. The C-index describes discrimination but cannot be converted reliably into sensitivity, specificity, or likelihood ratios at a particular decision threshold. This model was therefore retained in the systematic review but was not included in the sensitivity and specificity forest plots.

Reporting of calibration, threshold selection, and decision-analytic performance was inconsistent across the included reports. Discrimination alone does not establish whether predicted probabilities are accurate or whether model use would improve clinical decisions. Complete external-validation reporting should include the model equation or implementation details, prespecified thresholds, 2 x 2 contingency data, discrimination, calibration, and clinically relevant measures of net benefit.

Clinical interpretation

The model-specific sensitivity, specificity, and likelihood ratios describe diagnostic performance based on the extracted or reconstructed validation 2x2 tables. The four Li QH et al. models used study-reported thresholds in validation. For the Meng et al. model, the reported cutoff was 0.657, but it was not explicitly linked to the external-validation estimates; consequently, the reconstructed 2×2 results and derived likelihood ratios should be interpreted as approximate and cannot be attributed unequivocally to that cutoff. These measures should not be interpreted as pooled performance or as evidence that the models are interchangeable. No pooled likelihood ratios or pooled Fagan analysis were calculated because the available models represented different prediction tools, threshold information, comparator spectra, and clinical contexts.

Clinical utility extends beyond diagnostic accuracy. A model may show favorable sensitivity or specificity yet fail to improve decisions if it is poorly calibrated, difficult to implement, or evaluated in a population that differs from the intended target population. Evidence of clinical utility would require evaluation of calibration, decision-curve net benefit, effects on testing or treatment decisions, feasibility, resource use, and patient outcomes. The available evidence did not establish that implementation of any included model improves these outcomes.

Accordingly, the models should not yet be used as stand-alone tools for treatment initiation, isolation, or exclusion of BN-PTB. Their estimates may inform future validation studies, but implementation requires independent evaluation at an explicitly defined clinical use point and in a population that represents the intended diagnostic pathway.

Strengths and limitations

This review was registered in PROSPERO, searched four major bibliographic databases, retained all six eligible models rather than selecting a post hoc best-performing model, and distinguished reports, models, and validation samples as separate analytical units. The revised analysis presents model-specific performance and avoids pooling clinically different models or correlated estimates from a shared validation cohort. The PROBAST and GRADE-DTA assessments also underwent independent second-assessor review.

Several limitations should be acknowledged. First, only three reports were included, and four of the six models were evaluated in the same validation cohort. No model had sufficient independent external validations in comparable populations to support a same-model meta-analysis. Second, the comparator populations differed substantially across reports, limiting a direct comparison of specificity and likelihood ratios. Third, five models provided external-validation data sufficient for extraction or reconstruction of model-specific 2×2 tables, but only the four Li QH et al. models had study-reported thresholds explicitly applied in validation. For the Meng et al. model, the reported cutoff was 0.657, but it was not explicitly linked to the external-validation estimates; consequently, the reconstructed 2×2 results and derived likelihood ratios should be interpreted as approximate and cannot be attributed unequivocally to that cutoff. The remaining model reported an external C-index only. Fourth, calibration and decision-analytic measures were not reported consistently, and the available evidence did not include prospective clinical-impact evaluations. Nominal external-validation performance may also remain optimistic when development and validation cohorts share similar case mix, measurement procedures, referral mechanisms, or selection processes; the present review did not directly test these mechanisms. Fifth, the search did not include study registers, and unpublished or ongoing validation studies may therefore have been missed. Finally, the small number of reports and dependence among several model estimates precluded meta-regression, formal assessment of publication bias, and a meaningful quantitative synthesis.

Future research priorities

Future studies should prioritize repeated external validation of the same model in independent, consecutive, and clinically representative cohorts. The intended clinical role and use point should be specified before validation, including the stage of the diagnostic pathway at which the prediction is made and the decision that the model is intended to support.

Validation cohorts should use harmonized definitions of BN-PTB, clinically relevant non-tuberculosis comparators, and reference standards applied consistently across participants. Studies should report the complete model specification, rationale for threshold selection, threshold-specific true-positive, false-negative, true-negative, and false-positive counts, as well as discrimination and calibration measures. Decision-curve analysis and prospective impact studies are also needed to determine whether model-assisted care provides greater net benefit than existing diagnostic practice.

Head-to-head validation of multiple models in the same independent dataset would permit more reliable comparisons than comparisons across separate studies with different case mixes. When sufficient independent validations of the same model become available, model-specific meta-analysis or individual participant data meta-analysis may be appropriate. Until then, descriptive model-level synthesis provides a more defensible representation of the available evidence than cross-model pooling.

## Conclusions

Among the external-validation evidence meeting this review's completed eligibility criteria, three reports contributed six distinct BN-PTB prediction models. Five models provided data sufficient for extraction or reconstruction of model-specific 2×2 tables. The four Li QH et al. models had study-reported thresholds applied in validation; for the Meng et al. model, the reported cutoff was 0.657, but its explicit linkage to the external-validation estimates was unclear. Consequently, the reconstructed Meng 2×2 table and derived likelihood ratios should be considered approximate and cannot be attributed unequivocally to that cutoff. Although several models showed favorable model-specific performance, four estimates came from one shared validation cohort, comparator spectra differed across reports, and no model had repeated independent external validation in comparable populations. The available evidence therefore does not establish comparative performance, transportability, or clinical utility. Independent prospective multicenter validation of the same models in consecutive, clinically representative cohorts, with harmonized reference standards, prespecified thresholds, complete threshold-specific and calibration reporting, decision-curve analysis, and clinical-impact evaluation, is required before routine implementation.

## Appendices

Detailed electronic search strategies for PubMed, Embase, Web of Science, and Scopus are provided below. Searches were executed on March 7, 2026, and covered each database from inception through December 31, 2025. No study registers were searched.

**Table 3:** Bibliographic databases, search execution date, and coverage PubMed, Embase, Web of Science, and Scopus were searched on March 7, 2026, covering records from database inception through December 31, 2025. Complete database-specific search strategies are provided in the text immediately following this table. No study registers were searched. BN-PTB, bacteriologically negative pulmonary tuberculosis.

Database	Search execution date	Coverage
PubMed	March 7, 2026	Database inception through December 31, 2025
Embase	March 7, 2026	Database inception through December 31, 2025
Web of Science	March 7, 2026	Database inception through December 31, 2025
Scopus	March 7, 2026	Database inception through December 31, 2025

PubMed

Date range: Database inception through December 31, 2025

Full search strategy:

((((Pulmonary Tuberculosis OR "Pulmonary Consumption" OR "Consumption, Pulmonary" OR "Consumptions, Pulmonary" OR "Pulmonary Consumptions" OR "Pulmonary Phthisis" OR "Phthises, Pulmonary" OR "Phthisis, Pulmonary" OR "Pulmonary Phthises" OR "Pulmonary Tuberculoses" OR "Tuberculoses, Pulmonary" OR "Lung Tuberculosis" OR Tuberculoses OR "Koch's Disease" OR "Koch Disease" OR "Kochs Disease" OR "Mycobacterium tuberculosis Infection" OR "Infection, Mycobacterium tuberculosis" OR "Infections, Mycobacterium tuberculosis" OR "Mycobacterium tuberculosis Infections"))) AND (((Tuberculosis, Smear-Negative OR "smear negative" OR "sputum smear negative" OR "AFB negative" OR "acid-fast bacilli negative" OR "acid fast bacilli negative" OR "culture negative" OR "bacteriologically negative" OR "bacteriologically unconfirmed" OR "microbiologically negative" OR "microbiologically unconfirmed" OR "pathogen negative" OR "Xpert negative" OR "GeneXpert negative" OR "PCR negative" OR SNPT OR BN-PTB OR BNPT))) AND (((Machine Learning OR Artificial Intelligence OR Prediction Algorithms OR "Deep Learning" OR "Prediction Algorithm" OR "Predictive Algorithm" OR "Predictive Algorithms" OR "Predictive Model" OR "Predictive Models" OR "Diagnostic Model" OR "Diagnostic Models" OR "Risk Score" OR "Risk Scores" OR Algorithm OR Algorithms OR "Support Vector Machine" OR "Support Vector Machines" OR "Neural Network" OR "Neural Networks" OR "Random Forest" OR "Decision Tree" OR "Decision Trees" OR XGBoost OR Lasso OR "Elastic Net" OR "logistic regression" OR "Cox regression" OR AI OR ML))) AND (((Diagnostic Accuracy OR Sensitivity and Specificity OR ROC Curve OR "diagnostic performance" OR "diagnostic value" OR sensitivity OR specificity OR "predictive value" OR ROC OR Receiver Operating Characteristic OR AUC OR "Area Under the Curve" OR AUPRC OR "Precision-Recall" OR "Precision Recall" OR c-statistic OR "c statistic" OR c-index OR Calibration OR Hosmer-Lemeshow OR "Decision Curve Analysis" OR "Decision curve" OR "Net Benefit" OR NRI OR "Net Reclassification Improvement" OR IDI OR "Integrated Discrimination Improvement"))))

Embase

Date range: Database inception through December 31, 2025

Full search strategy:

('pulmonary consumption' OR 'consumption, pulmonary' OR 'consumptions, pulmonary' OR 'pulmonary consumptions' OR 'pulmonary phthisis'/exp OR 'pulmonary phthisis' OR 'phthises, pulmonary' OR 'phthisis, pulmonary' OR 'pulmonary phthises' OR 'pulmonary tuberculoses' OR 'pulmonary tuberculosis'/exp OR 'pulmonary tuberculosis' OR 'tuberculoses, pulmonary' OR 'lung tuberculosis'/exp OR 'lung tuberculosis' OR 'tuberculoses' OR 'koch disease' OR 'kochs disease' OR 'mycobacterium tuberculosis infection'/exp OR 'mycobacterium tuberculosis infection' OR 'infection, mycobacterium tuberculosis' OR 'infections, mycobacterium tuberculosis' OR 'mycobacterium tuberculosis infections') AND ('tuberculosis, smear-negative' OR 'smear negative' OR 'sputum smear negative' OR 'afb negative' OR 'acid-fast bacilli negative' OR 'acid fast bacilli negative' OR 'culture negative' OR 'bacteriologically negative' OR 'bacteriologically unconfirmed' OR 'microbiologically negative' OR 'microbiologically unconfirmed' OR 'pathogen negative' OR 'xpert negative' OR 'genexpert negative' OR 'pcr negative' OR 'snpt' OR 'bn-ptb' OR 'bnpt') AND ('machine learning'/exp OR 'machine learning' OR 'artificial intelligence'/exp OR 'artificial intelligence' OR 'prediction algorithms'/exp OR 'prediction algorithms' OR 'deep learning'/exp OR 'deep learning' OR 'prediction algorithm'/exp OR 'prediction algorithm' OR 'predictive algorithm'/exp OR 'predictive algorithm' OR 'predictive algorithms' OR 'predictive model'/exp OR 'predictive model' OR 'predictive models' OR 'diagnostic model'/exp OR 'diagnostic model' OR 'diagnostic models' OR 'risk score'/exp OR 'risk score' OR 'risk scores' OR 'algorithm'/exp OR 'algorithm' OR 'algorithms'/exp OR 'algorithms' OR 'support vector machine'/exp OR 'support vector machine' OR 'support vector machines'/exp OR 'support vector machines' OR 'neural network'/exp OR 'neural network' OR 'neural networks'/exp OR 'neural networks' OR 'random forest'/exp OR 'random forest' OR 'decision tree'/exp OR 'decision tree' OR 'decision trees'/exp OR 'decision trees' OR 'xgboost'/exp OR 'xgboost' OR 'lasso'/exp OR 'lasso' OR 'elastic net'/exp OR 'elastic net' OR 'logistic regression'/exp OR 'logistic regression' OR 'cox regression'/exp OR 'cox regression' OR 'ai' OR 'ml') AND ('diagnostic accuracy'/exp OR 'diagnostic accuracy' OR 'sensitivity and specificity'/exp OR 'sensitivity and specificity' OR 'roc curve'/exp OR 'roc curve' OR 'diagnostic performance'/exp OR 'diagnostic performance' OR 'diagnostic value'/exp OR 'diagnostic value' OR 'sensitivity'/exp OR 'sensitivity' OR 'specificity'/exp OR 'specificity' OR 'predictive value'/exp OR 'predictive value' OR 'roc' OR 'receiver operating characteristic'/exp OR 'receiver operating characteristic' OR 'auc'/exp OR 'auc' OR 'area under the curve'/exp OR 'area under the curve' OR 'auprc' OR 'precision-recall' OR 'precision recall' OR 'c-statistic' OR 'c statistic'/exp OR 'c statistic' OR 'c-index' OR 'calibration'/exp OR 'calibration' OR 'hosmer-lemeshow' OR 'decision curve analysis'/exp OR 'decision curve analysis' OR 'decision curve'/exp OR 'decision curve' OR 'net benefit' OR 'nri' OR 'net reclassification improvement'/exp OR 'net reclassification improvement' OR 'idi' OR 'integrated discrimination improvement'/exp OR 'integrated discrimination improvement') 

Date filter applied in Embase: publication years 1000 through 2025 (py field).

Web of Science

Date range: Database inception through December 31, 2025

Full search strategy:

(TS=("Pulmonary Consumption" OR "Consumption, Pulmonary" OR "Consumptions, Pulmonary" OR "Pulmonary Consumptions" OR "Pulmonary Phthisis" OR "Phthises, Pulmonary" OR "Phthisis, Pulmonary" OR "Pulmonary Phthises" OR "Pulmonary Tuberculoses" OR "Pulmonary Tuberculosis" OR "Tuberculoses, Pulmonary" OR "Lung Tuberculosis" OR "Tuberculoses" OR "Koch Disease" OR "Kochs Disease" OR "Mycobacterium tuberculosis Infection" OR "Infection, Mycobacterium tuberculosis" OR "Infections, Mycobacterium tuberculosis" OR "Mycobacterium tuberculosis Infections")) AND (TS=("Tuberculosis, Smear-Negative" OR "smear negative" OR "sputum smear negative" OR "AFB negative" OR "acid-fast bacilli negative" OR "acid fast bacilli negative" OR "culture negative" OR "bacteriologically negative" OR "bacteriologically unconfirmed" OR "microbiologically negative" OR "microbiologically unconfirmed" OR "pathogen negative" OR "Xpert negative" OR "GeneXpert negative" OR "PCR negative" OR "SNPT" OR "BN-PTB" OR "BNPT")) AND (TS=("Machine Learning" OR "Artificial Intelligence" OR "Prediction Algorithms" OR "Deep Learning" OR "Prediction Algorithm" OR "Predictive Algorithm" OR "Predictive Algorithms" OR "Predictive Model" OR "Predictive Models" OR "Diagnostic Model" OR "Diagnostic Models" OR "Risk Score" OR "Risk Scores" OR "Algorithm" OR "Algorithms" OR "Support Vector Machine" OR "Support Vector Machines" OR "Neural Network" OR "Neural Networks" OR "Random Forest" OR "Decision Tree" OR "Decision Trees" OR "XGBoost" OR "Lasso" OR "Elastic Net" OR "logistic regression" OR "Cox regression" OR "AI" OR "ML")) AND (TS=("Diagnostic Accuracy" OR "Sensitivity and Specificity" OR "ROC Curve" OR "diagnostic performance" OR "diagnostic value" OR "sensitivity" OR "specificity" OR "predictive value" OR "ROC" OR "Receiver Operating Characteristic" OR "AUC" OR "Area Under the Curve" OR "AUPRC" OR "Precision-Recall" OR "Precision Recall" OR "c-statistic" OR "c statistic" OR "c-index" OR "Calibration" OR "Hosmer-Lemeshow" OR "Decision Curve Analysis" OR "Decision curve" OR "Net Benefit" OR "NRI" OR "Net Reclassification Improvement" OR "IDI" OR "Integrated Discrimination Improvement"))

Scopus

Date range: Database inception through December 31, 2025

Full search strategy:

TITLE-ABS-KEY(("Pulmonary Consumption" OR "Consumption, Pulmonary" OR "Consumptions, Pulmonary" OR "Pulmonary Consumptions" OR "Pulmonary Phthisis" OR "Phthises, Pulmonary" OR "Phthisis, Pulmonary" OR "Pulmonary Phthises" OR "Pulmonary Tuberculoses" OR "Pulmonary Tuberculosis" OR "Tuberculoses, Pulmonary" OR "Lung Tuberculosis" OR "Tuberculoses" OR "Koch Disease" OR "Kochs Disease" OR "Mycobacterium tuberculosis Infection" OR "Infection, Mycobacterium tuberculosis" OR "Infections, Mycobacterium tuberculosis" OR "Mycobacterium tuberculosis Infections") AND ("Tuberculosis, Smear-Negative" OR "smear negative" OR "sputum smear negative" OR "AFB negative" OR "acid-fast bacilli negative" OR "acid fast bacilli negative" OR "culture negative" OR "bacteriologically negative" OR "bacteriologically unconfirmed" OR "microbiologically negative" OR "microbiologically unconfirmed" OR "pathogen negative" OR "Xpert negative" OR "GeneXpert negative" OR "PCR negative" OR "SNPT" OR "BN-PTB" OR "BNPT") AND ("Machine Learning" OR "Artificial Intelligence" OR "Prediction Algorithms" OR "Deep Learning" OR "Prediction Algorithm" OR "Predictive Algorithm" OR "Predictive Algorithms" OR "Predictive Model" OR "Predictive Models" OR "Diagnostic Model" OR "Diagnostic Models" OR "Risk Score" OR "Risk Scores" OR "Algorithm" OR "Algorithms" OR "Support Vector Machine" OR "Support Vector Machines" OR "Neural Network" OR "Neural Networks" OR "Random Forest" OR "Decision Tree" OR "Decision Trees" OR "XGBoost" OR "Lasso" OR "Elastic Net" OR "logistic regression" OR "Cox regression" OR "AI" OR "ML") AND ("Diagnostic Accuracy" OR "Sensitivity and Specificity" OR "ROC Curve" OR "diagnostic performance" OR "diagnostic value" OR "sensitivity" OR "specificity" OR "predictive value" OR "ROC" OR "Receiver Operating Characteristic" OR "AUC" OR "Area Under the Curve" OR "AUPRC" OR "Precision-Recall" OR "Precision Recall" OR "c-statistic" OR "c statistic" OR "c-index" OR "Calibration" OR "Hosmer-Lemeshow" OR "Decision Curve Analysis" OR "Decision curve" OR "Net Benefit" OR "NRI" OR "Net Reclassification Improvement" OR "IDI" OR "Integrated Discrimination Improvement"))

**Table 4:** Source-supported rationales for the PROBAST assessments of included reports Source-supported rationales underlying the domain-level PROBAST risk-of-bias judgments for the six prediction models reported across three included reports: Li QH et al. [26], Meng et al. [10], and Xu et al. [18]. Each report is summarized across the participants, predictors, outcome, and analysis domains. The Li QH et al. report contributed four models (CR, CRE, CRP, and CREP), whereas the Meng et al. and Xu et al. reports each contributed one model. Development and external-validation evaluations were assessed separately in the complete PROBAST signaling-question assessment. AFB-/IGRA+, acid-fast bacilli smear-negative and interferon-gamma release assay-positive; AIC, Akaike information criterion; BN-PTB, bacteriologically negative pulmonary tuberculosis; CR, clinical-radiographic; CRE, clinical-radiographic-radionuclide; CRP, clinical-radiographic-pathological; CREP, clinical-radiographic-radionuclide-pathological; DCA, decision-curve analysis; ECT, emission computed tomography; LASSO, least absolute shrinkage and selection operator; PROBAST, Prediction model Risk Of Bias ASsessment Tool; PTB, pulmonary tuberculosis; TB, tuberculosis; TST, tuberculin skin test; WBC, white blood cell count.

Report	PROBAST domain	Source-supported rationale
Li QH et al. [26]	Participants	The development sample was a retrospective, biopsy-based comparison of known sarcoidosis and sputum-negative TB cases. The validation sample was a later set of 73 sarcoidosis and 57 TB cases rather than a consecutive cohort of diagnostically uncertain patients.
Li QH et al. [26]	Predictors	Clinical, radiographic, radionuclide, and histopathologic data were abstracted retrospectively. Blinding of radiology/pathology assessment to final diagnosis was not reported. CRE/CREP require ECT; CRP/CREP require biopsy-derived variables.
Li QH et al. [26]	Outcome	TB and sarcoidosis were established through different combinations of histology and treatment response. Necrosis, used in CRP and CREP, was also part of the TB inclusion/reference definition. Outcome assessment blinding was not reported.
Li QH et al. [26]	Analysis	Candidate variables were screened by univariable chi-square tests and backward logistic regression; TST cutoff and model cutoffs were selected from the development data. Missingness was handled by complete-case analysis and a missing-indicator approach. Calibration and optimism adjustment were not reported. The CREP table reports a beta coefficient of -0.85 for increased reticular fiber but assigns a positive score of 1, so correspondence of the final score to the multivariable coefficient is not demonstrable.
Meng et al. [10]	Participants	The derivation cohort was prospectively and consecutively recruited among adults with suspected PTB and negative smear, culture, and PCR. The independent temporal validation cohort used a similar strategy but added 50 etiologically confirmed PTB cases, creating a target-population mismatch for BN-PTB validation.
Meng et al. [10]	Predictors	Predictors were collected on admission using stated laboratory procedures; imaging was evaluated by radiologists. The model retained seven predictors and reported the full logistic equation and cutoff.
Meng et al. [10]	Outcome	Two pulmonologists separately assigned clinical diagnoses using WS 288-2017, but the paper did not report blinding to predictor information. The clinical standard likely used symptoms, imaging, and laboratory information that overlap with model predictors. Validation mixed etiological confirmation and clinical diagnosis.
Meng et al. [10]	Analysis	Thirty-five candidate variables were considered with 111 clinically diagnosed PTB outcomes in development. LASSO, logistic regression, and 10-fold cross-validation were reported, but missing-data handling was not described. Validation included 127 PTB and 140 non-TB controls and reported discrimination, calibration curves, and DCA; missing-data handling remained unreported.
Xu et al. [18]	Participants	The retrospective discovery data began with much larger TB and pneumonia groups, but only 89 AFB-/IGRA+ TB and 38 pneumonia participants with biomarker values entered model development after missing-data exclusions. Fifteen of 134 external participants were also excluded for missing variables.
Xu et al. [18]	Predictors	Routine laboratory predictors were log2-transformed and available at the diagnostic assessment. Their measurement is largely objective, although predictor-assessment blinding was not explicitly reported.
Xu et al. [18]	Outcome	AFB-negative TB was defined using imaging and treatment response, whereas pneumonia followed a separate national standard. WBC was both a model predictor and an element of the pneumonia diagnostic definition. Outcome assessment without predictor knowledge was not reported.
Xu et al. [18]	Analysis	Forty-one biomarkers were screened using differential testing and univariable logistic regression, then an AIC stopping rule selected the multivariable model. Complete-case exclusion was used rather than imputation. The development and validation samples were small, and the full model coefficients were not reported. Bootstrap calibration was reported, but it did not resolve the selection, missing-data, and sample-size concerns.

**Table 5:** Complete PROBAST signaling-question assessments for included models Complete PROBAST signaling-question judgments for six prediction models and 12 evaluation units. Each model column reports paired development/external-validation judgments in D/V order. Questions not applicable to external validation are marked NA. CR, clinical-radiographic; CRE, clinical-radiographic-radionuclide; CRP, clinical-radiographic-pathological; CREP, clinical-radiographic-radionuclide-pathological; D, development; N, no; NA, not applicable; NI, no information; PN, probably no; PROBAST, Prediction model Risk Of Bias ASsessment Tool; PY, probably yes; RCT, randomized controlled trial; V, external validation; Y, yes.

Signaling question and official wording	Li CR D / V	Li CRE D / V	Li CRP D / V	Li CREP D / V	Meng LASSO-logistic D / V	Xu nomogram D / V
1.1 Were appropriate data sources used, e.g., cohort, RCT, or nested case-control study data?	N / N	N / N	N / N	N / N	Y / Y	N / N
1.2 Were all inclusions and exclusions of participants appropriate?	PN / PN	PN / PN	PN / PN	PN / PN	PY / PY	N / N
2.1 Were predictors defined and assessed in a similar way for all participants?	PY / NI	PY / NI	PY / NI	PY / NI	Y / Y	PY / PY
2.2 Were predictor assessments made without knowledge of outcome data?	N / NI	N / NI	N / NI	N / NI	PY / PY	PY / PY
2.3 Are all predictors available at the time the model is intended to be used?	Y / Y	Y / Y	Y / Y	Y / Y	Y / Y	Y / Y
3.1 Was the outcome determined appropriately?	PY / PY	PY / PY	PY / PY	PY / PY	PY / PY	PN / PN
3.2 Was a prespecified or standard outcome definition used?	PY / PY	PY / PY	PY / PY	PY / PY	Y / Y	PY / PY
3.3 Were predictors excluded from the outcome definition?	Y / Y	Y / Y	N / N	N / N	PN / PN	N / N
3.4 Was the outcome defined and determined in a similar way for all participants?	N / N	N / N	N / N	N / N	PY / N	N / N
3.5 Was the outcome determined without knowledge of predictor information?	N / N	N / N	N / N	N / N	PN / PN	N / N
3.6 Was the time interval between predictor assessment and outcome determination appropriate?	NI / NI	NI / NI	NI / NI	NI / NI	PY / PY	NI / NI
4.1 Were there a reasonable number of participants with the outcome?	N / N	N / N	N / N	N / N	N / Y	N / N
4.2 Were continuous and categorical predictors handled appropriately?	N / Y	N / Y	N / Y	N / Y	Y / Y	PY / PY
4.3 Were all enrolled participants included in the analysis?	NI / NI	NI / NI	NI / NI	NI / NI	NI / Y	N / N
4.4 Were participants with missing data handled appropriately?	N / NI	N / NI	N / NI	N / NI	NI / NI	N / N
4.5 Was selection of predictors based on univariable analysis avoided?	N / NA	N / NA	N / NA	N / NA	Y / NA	N / NA
4.6 Were complexities in the data, including sampling of controls, accounted for appropriately?	N / N	N / N	N / N	N / N	Y / Y	N / N
4.7 Were relevant model performance measures evaluated appropriately?	N / N	N / N	N / N	N / N	PY / PY	PY / PY
4.8 Were model overfitting, underfitting, and optimism in model performance accounted for?	N / NA	N / NA	N / NA	N / NA	PY / NA	PN / NA
4.9 Do predictors and their assigned weights in the final model correspond to the reported multivariable analysis?	PY / NA	PY / NA	PY / NA	N / NA	Y / NA	NI / NA

**Table 6:** Model- and outcome-specific GRADE-DTA evidence profile Certainty of evidence was assessed separately for each reported model-outcome using an adaptation of GRADE-DTA. Each evidence body contained one external-validation evaluation. [a] Risk of bias was rated very serious because the corresponding external-validation evaluation was at high overall risk of bias. [b] Inconsistency across studies was not assessable because each model-outcome evidence body contained only one evaluation. [c] Indirectness was rated very serious for Li and Xu and serious for Meng, consistent with the applicability limitations summarized in Tables 2 and 4. [d] Imprecision was rated serious because each outcome was supported by one limited validation sample without repeated validation in comparable settings. [e] Publication bias was not assessable because a single evaluation cannot establish the presence or absence of bias from unpublished whole studies. Overall certainty remained very low after downgrading for risk of bias, indirectness, and imprecision. Decision-curve analyses and calibration plots were not treated as direct evidence of improved patient-important outcomes, and no prospective clinical-impact study was identified. GRADE-DTA, Grading of Recommendations Assessment, Development and Evaluation for diagnostic test accuracy; ROB, risk of bias.

Model and outcome	Evidence	ROB[a]	Inconsistency[b]	Indirectness[c]	Imprecision[d]	Publication bias[e]	Certainty
Li CR external validation: sensitivity	1 evaluation; 130 participants	Very serious	Not assessable across studies	Very serious	Serious	Not assessable	Very low
Li CR external validation: specificity	1 evaluation; 130 participants	Very serious	Not assessable across studies	Very serious	Serious	Not assessable	Very low
Li CRE external validation: sensitivity	1 evaluation; 130 participants	Very serious	Not assessable across studies	Very serious	Serious	Not assessable	Very low
Li CRE external validation: specificity	1 evaluation; 130 participants	Very serious	Not assessable across studies	Very serious	Serious	Not assessable	Very low
Li CRP external validation: sensitivity	1 evaluation; 130 participants	Very serious	Not assessable across studies	Very serious	Serious	Not assessable	Very low
Li CRP external validation: specificity	1 evaluation; 130 participants	Very serious	Not assessable across studies	Very serious	Serious	Not assessable	Very low
Li CREP external validation: sensitivity	1 evaluation; 130 participants	Very serious	Not assessable across studies	Very serious	Serious	Not assessable	Very low
Li CREP external validation: specificity	1 evaluation; 130 participants	Very serious	Not assessable across studies	Very serious	Serious	Not assessable	Very low
Meng LASSO-logistic external validation: sensitivity	1 evaluation; 267 participants	Very serious	Not assessable across studies	Serious	Serious	Not assessable	Very low
Meng LASSO-logistic external validation: specificity	1 evaluation; 267 participants	Very serious	Not assessable across studies	Serious	Serious	Not assessable	Very low
Xu nomogram external validation: C-index	1 evaluation; 119 analyzed participants	Very serious	Not assessable across studies	Very serious	Serious	Not assessable	Very low
